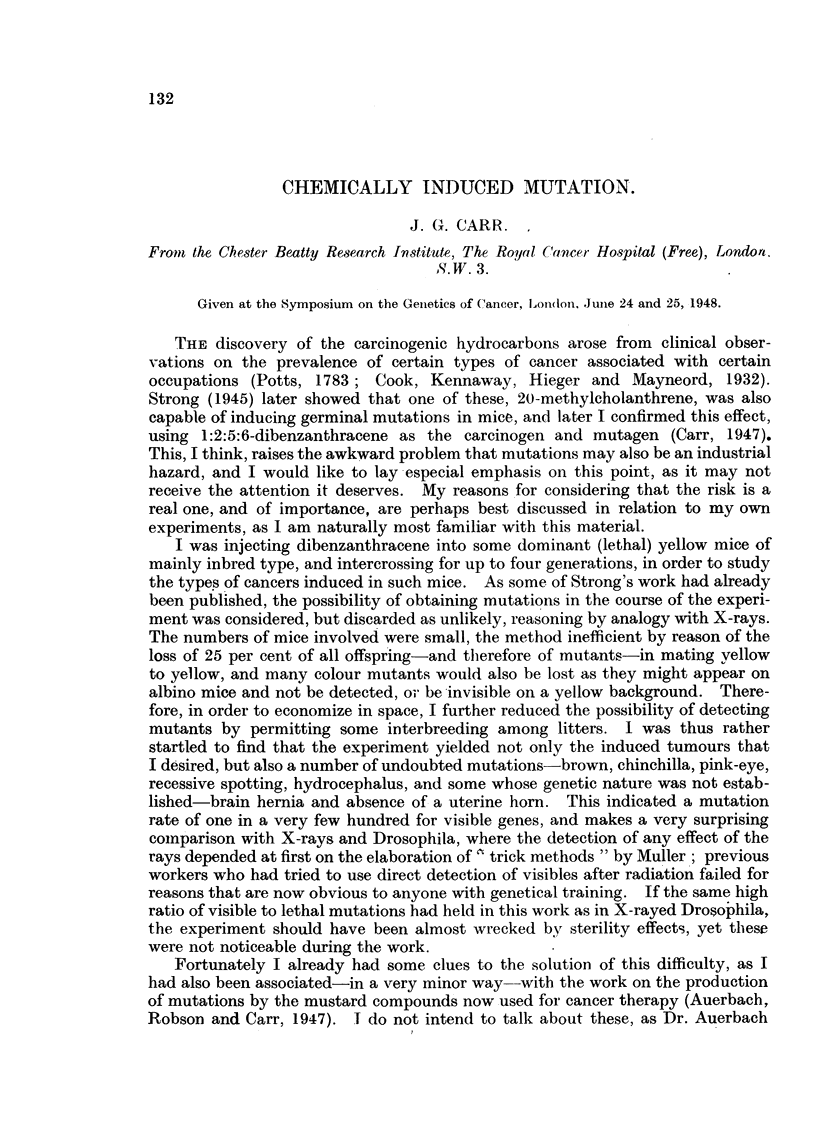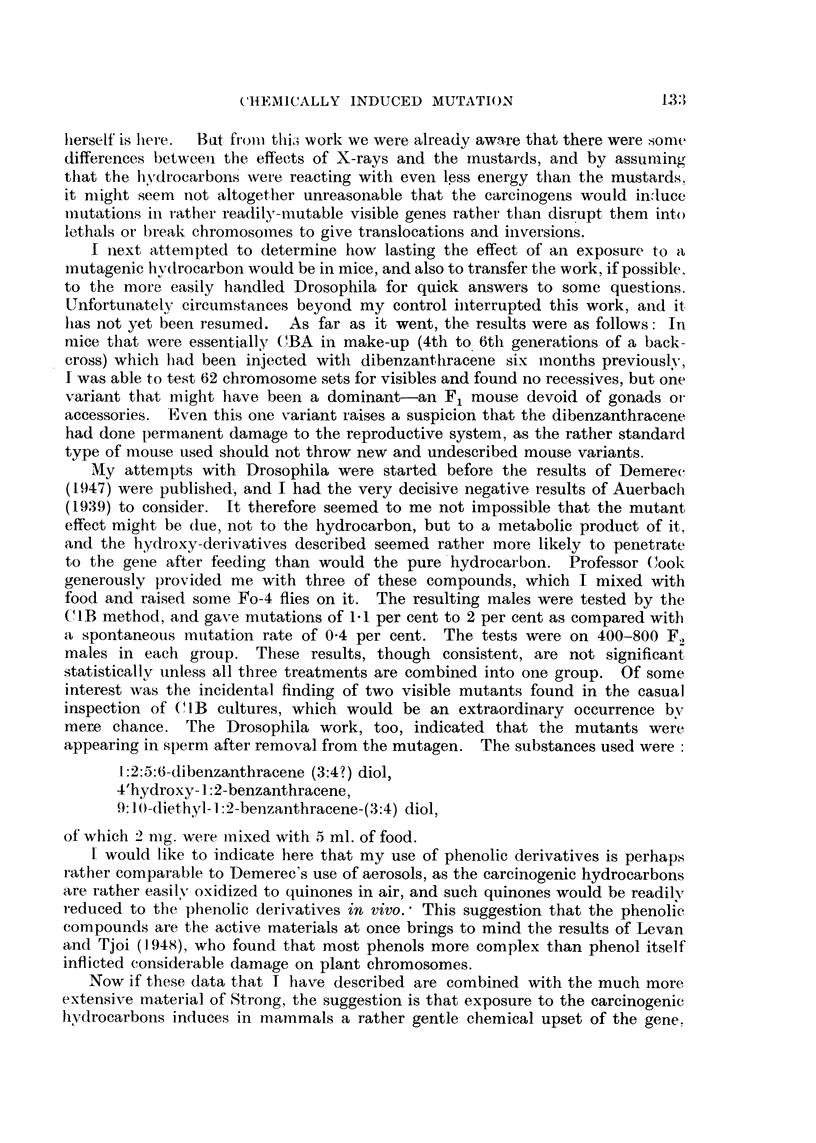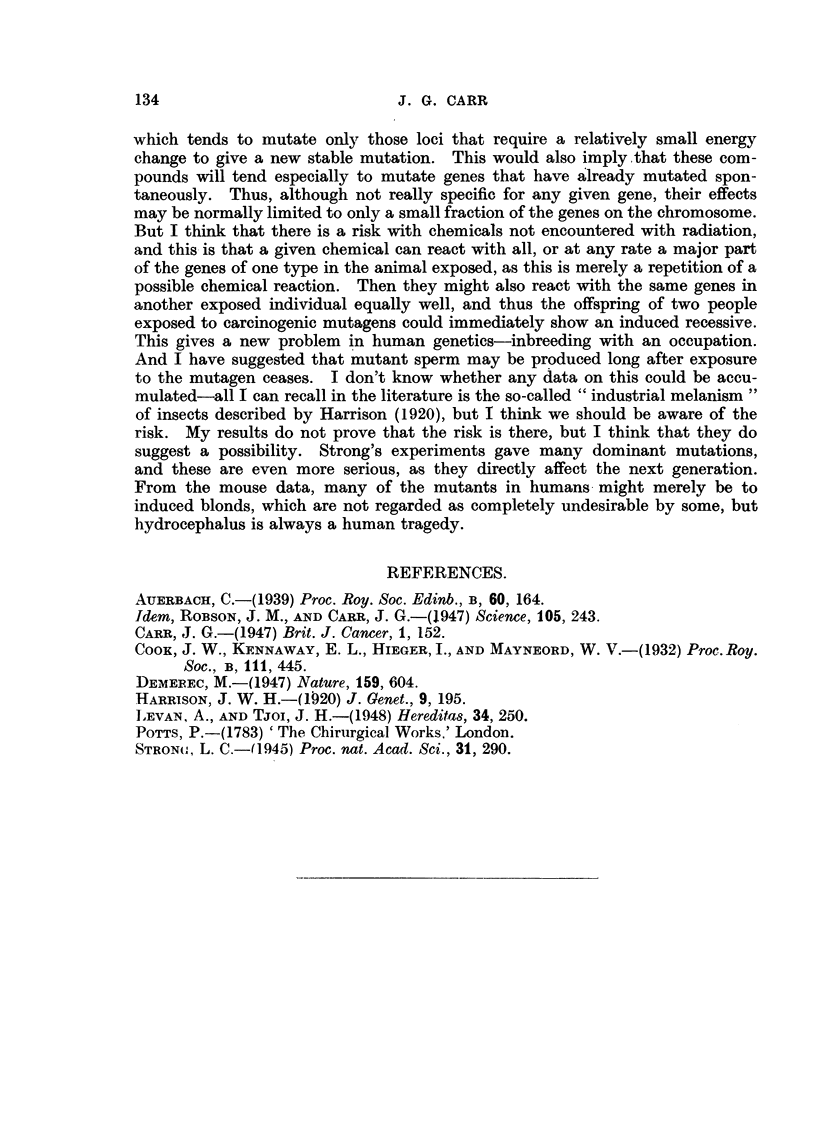# Chemically Induced Mutation

**DOI:** 10.1038/bjc.1948.19

**Published:** 1948-06

**Authors:** J. G. Carr


### CHEMICALLY INDUCED MUTATION.

J. G. CARR.

*From the Chester Beatty Research Institute, The Royal Cancer Hospital (Free), London, S.W. 3.*

Given at the Symposium on the Genetics of Cancer, London, June 24 and 25, 1948.

THE discovery of the carcinogenic hydrocarbons arose from clinical observations on the prevalence of certain types of cancer associated with certain occupations (Potts, 1783 ; Cook, Kennaway, Hieger and Mayneord, 1932). Strong (1945) later showed that one of these, 20-methylcholanthrene, was also capable of inducing germinal mutations in mice, and later I confirmed this effect, using 1:2:5:6-dibenzanthracene as the carcinogen and mutagen (Carr, 1947). This, I think, raises the awkward problem that mutations may also be an industrial hazard, and I would like to lay especial emphasis on this point, as it may not receive the attention it deserves. My reasons for considering that the risk is a real one, and of importance, are perhaps best discussed in relation to my own experiments, as I am naturally most familiar with this material.

I was injecting dibenzanthracene into some dominant (lethal) yellow mice of mainly inbred type, and intercrossing for up to four generations, in order to study the types of cancers induced in such mice. As some of Strong's work had already been published, the possibility of obtaining mutations in the course of the experiment was considered, but discarded as unlikely, reasoning by analogy with X-rays. The numbers of mice involved were small, the method inefficient by reason of the loss of 25 per cent of all offspring—and therefore of mutants—in mating yellow to yellow, and many colour mutants would also be lost as they might appear on albino mice and not be detected, or be invisible on a yellow background. Therefore, in order to economize in space, I further reduced the possibility of detecting mutants by permitting some interbreeding among litters. I was thus rather startled to find that the experiment yielded not only the induced tumours that I desired, but also a number of undoubted mutations—brown, chinchilla, pink-eye, recessive spotting, hydrocephalus, and some whose genetic nature was not established—brain hernia and absence of a uterine horn. This indicated a mutation rate of one in a very few hundred for visible genes, and makes a very surprising comparison with X-rays and Drosophila, where the detection of any effect of the rays depended at first on the elaboration of " trick methods " by Muller ; previous workers who had tried to use direct detection of visibles after radiation failed for reasons that are now obvious to anyone with genetical training. If the same high ratio of visible to lethal mutations had held in this work as in X-rayed Drosophila, the experiment should have been almost wrecked by sterility effects, yet these were not noticeable during the work.

Fortunately I already had some clues to the solution of this difficulty, as I had also been associated—in a very minor way—with the work on the production of mutations by the mustard compounds now used for cancer therapy (Auerbach, Robson and Carr, 1947). I do not intend to talk about these, as Dr. Auerbach herself is here. But from this work we were already aware that there were some differences between the effects of X-rays and the mustards, and by assuming that the hydrocarbons were reacting with even less energy than the mustards, it might seem not altogether unreasonable that the carcinogens would induce mutations in rather readily-mutable visible genes rather than disrupt them into lethals or break chromosomes to give translocations and inversions.

I next attempted to determine how lasting the effect of an exposure to a mutagenic hydrocarbon would be in mice, and also to transfer the work, if possible, to the more easily handled Drosophila for quick answers to some questions. Unfortunately circumstances beyond my control interrupted this work, and it has not yet been resumed. As far as it went, the results were as follows: In mice that were essentially CBA in make-up (4th to 6th generations of a back-cross) which had been injected with dibenzanthracene six months previously, I was able to test 62 chromosome sets for visibles and found no recessives, but one variant that might have been a dominant—an $F_1$ mouse devoid of gonads or accessories. Even this one variant raises a suspicion that the dibenzanthracene had done permanent damage to the reproductive system, as the rather standard type of mouse used should not throw new and undescribed mouse variants.

My attempts with Drosophila were started before the results of Demerec (1947) were published, and I had the very decisive negative results of Auerbach (1939) to consider. It therefore seemed to me not impossible that the mutant effect might be due, not to the hydrocarbon, but to a metabolic product of it, and the hydroxy-derivatives described seemed rather more likely to penetrate to the gene after feeding than would the pure hydrocarbon. Professor Cook generously provided me with three of these compounds, which I mixed with food and raised some Fo-4 flies on it. The resulting males were tested by the ClB method, and gave mutations of 1·1 per cent to 2 per cent as compared with a spontaneous mutation rate of 0·4 per cent. The tests were on 400–800 $F_2$ males in each group. These results, though consistent, are not significant statistically unless all three treatments are combined into one group. Of some interest was the incidental finding of two visible mutants found in the casual inspection of ClB cultures, which would be an extraordinary occurrence by mere chance. The Drosophila work, too, indicated that the mutants were appearing in sperm after removal from the mutagen. The substances used were :

1:2:5:6-dibenzanthracene (3:4?) diol,
4'hydroxy-1:2-benzanthracene,
9:10-diethyl-1:2-benzanthracene-(3:4) diol, of which 2 mg. were mixed with 5 ml. of food.

I would like to indicate here that my use of phenolic derivatives is perhaps rather comparable to Demerec's use of aerosols, as the carcinogenic hydrocarbons are rather easily oxidized to quinones in air, and such quinones would be readily reduced to the phenolic derivatives *in vivo.* This suggestion that the phenolic compounds are the active materials at once brings to mind the results of Levan and Tjoi (1948), who found that most phenols more complex than phenol itself inflicted considerable damage on plant chromosomes.

Now if these data that I have described are combined with the much more extensive material of Strong, the suggestion is that exposure to the carcinogenic hydrocarbons induces in mammals a rather gentle chemical upset of the gene, which tends to mutate only those loci that require a relatively small energy change to give a new stable mutation. This would also imply that these compounds will tend especially to mutate genes that have already mutated spontaneously. Thus, although not really specific for any given gene, their effects may be normally limited to only a small fraction of the genes on the chromosome. But I think that there is a risk with chemicals not encountered with radiation, and this is that a given chemical can react with all, or at any rate a major part of the genes of one type in the animal exposed, as this is merely a repetition of a possible chemical reaction. Then they might also react with the same genes in another exposed individual equally well, and thus the offspring of two people exposed to carcinogenic mutagens could immediately show an induced recessive. This gives a new problem in human genetics—inbreeding with an occupation. And I have suggested that mutant sperm may be produced long after exposure to the mutagen ceases. I don't know whether any data on this could be accumulated—all I can recall in the literature is the so-called " industrial melanism " of insects described by Harrison (1920), but I think we should be aware of the risk. My results do not prove that the risk is there, but I think that they do suggest a possibility. Strong's experiments gave many dominant mutations, and these are even more serious, as they directly affect the next generation. From the mouse data, many of the mutants in humans might merely be to induced blonds, which are not regarded as completely undesirable by some, but hydrocephalus is always a human tragedy.

### REFERENCES.

AUERBACH, C.—(1939) *Proc. Roy. Soc. Edinb.*, B, **60**, 164.

*Idem*, ROBSON, J. M., AND CARR, J. G.—(1947) *Science*, **105**, 243.

CARR, J. G.—(1947) *Brit. J. Cancer*, **1**, 152.

COOK, J. W., KENNAWAY, E. L., HIEGER, I., AND MAYNEORD, W. V.—(1932) *Proc. Roy. Soc.*, B, **111**, 445.

DEMEREC, M.—(1947) *Nature*, **159**, 604.

HARRISON, J. W. H.—(1920) *J. Genet.*, **9**, 195.

LEVAN, A., AND TJOI, J. H.—(1948) *Hereditas*, **34**, 250.

POTTS, P.—(1783) 'The Chirurgical Works,' London.

STRONG, L. C.—(1945) *Proc. nat. Acad. Sci.*, **31**, 290.